# Rapamycin prevents the impairments of social recognition induced by anti-P antibody in a murine model

**DOI:** 10.1136/annrheumdis-2019-216563

**Published:** 2019-12-09

**Authors:** Xuejiao Wang, Pingting Yang, Ling Qin

**Affiliations:** 1 Department of Physiology, China Medical University, Shenyang, China; 2 Department of Rheumatology and Immunology, First Affiliated Hospital, China Medical University, Shenyang, China

**Keywords:** autoantibodies, systemic lupus erythematosus, autoimmune diseases

**Figure 1:**
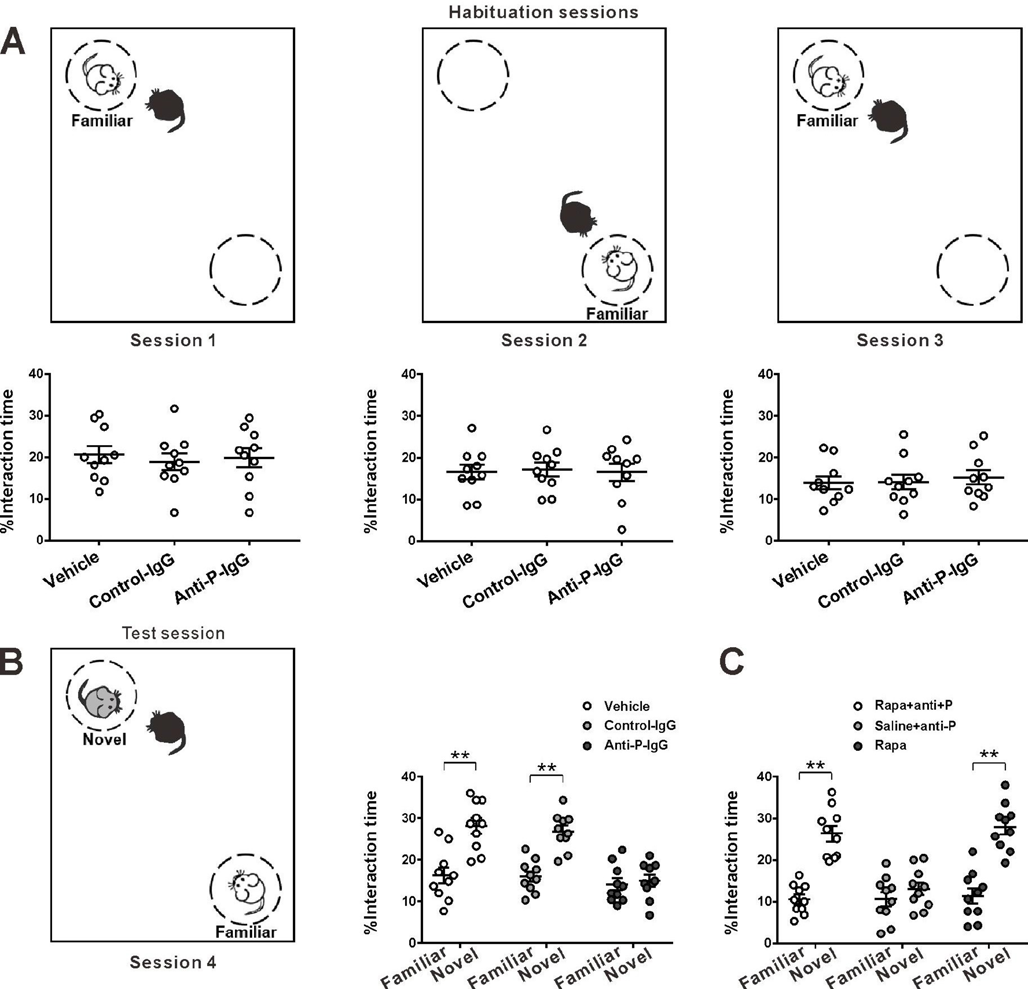
Behavioural schematic, and interaction time when the test mouse was interacting with the stimulus mice in habituation (A) and test sessions (B). Effect of pretreatment of rapamycin. (C) All data are displayed as mean±SEM; n=10. **P<0.01, t-test. anti-P, antiribosomal P.

The antiribosomal P antibody (anti-P) is detected predominantly in patients with systemic lupus erythematosus (SLE)^1^ and associated with a variety of neuropsychiatric manifestations (psychosis, mood disorders, cognitive decline, seizures and aseptic meningitis).^2 3^ A causal link between anti-P and the neuropsychiatric problems remains to be verified. Recently, anti-P has increasingly been associated with memory impairments. By passive transfer of anti-P into the brain of animals, previous experiments have revealed that hippocampus neurons are prime targets of anti-P, and spatial memory is impaired by the transferred anti-P.^4 5^ However, it remains unknown whether anti-P affects on the social memory (the memory of familiar conspecifics). Recently, the ventral CA1 region of hippocampus (vCA1) has been found to play a necessary and sufficient role in social memory.^6^ We, therefore, directly injected anti-P IgG (1.7 mg/mL, 0.5 µL) isolated from SLE patient sera, or control IgG from normal individuals or vehicle (artificial cerebrospinal fluid) into vCA1 of normal mice (see details in online supplementary text), and at 24 hours later, we used the social discrimination task to evaluate the impact of anti-P in social memory of mice. As shown in figure 1A, a test mouse was placed in a plexiglass arena, and two pencil-wire cups were placed on opposing corners (one was empty, the other enclosed a mouse). The test mouse habituated to the stimulus mouse during the first three sessions (5 min), rendering it ‘familiar’. During the fourth session, a novel mouse was placed in the opposing cup and the three mice were in the same arena. The subject was tested for discrimination between the novel and familiar mouse. Mice received vehicle or control IgG injection showed a longer duration for interaction to a novel mouse than to a familiar mouse, whereas anti-P-injected mice had no preference to a novel mouse, indicating an impairment of social memory (figure 1A, B). We also found that the olfactory and locomotor abilities were not altered in the mice (see online supplementary text and figures S1 and S2), suggesting that the anti-P injection did not cause sensory and motor deficits.

10.1136/annrheumdis-2019-216563.supp1 Supplementary data

10.1136/annrheumdis-2019-216563.supp2 Supplementary data

10.1136/annrheumdis-2019-216563.supp3 Supplementary data

Next, we used a new cohort of mice to examine whether application of rapamycin can protect against the anti-P-induced impairment. The mice were randomly divided into three groups: received daily intraperitoneal injection of rapamycin (0.1 mg/kg) for 7 days before anti-P injection (Rapa +anti-P), the same dose of saline injection and anti-P as the control group (Saline +anti-P), and rapamycin alone (Rapa). The mice of Rapa +anti-P showed a significant discrimination between the familiar and novel mice, while those of saline +anti-P group did not (figure 1C), suggesting that rapamycin can prevent the social memory impairment induced by anti-P. The application of rapamycin alone has no significant effect on social memory. Here, we present the first evidence showing a detrimental role of anti-P in social memory and the preventive effect of rapamycin. Further systemic experiments are warranted to examine whether and how rapamycin can rescue the autoantibody-induced impairments in patients.
