# Supplementary material for: Rapamycin prevents the impairments of social recognition induced by anti-P antibody in a murine model
Source: Ann Rheum Dis. 2019 Dec 9;79(3):428–9. doi: 10.1136/annrheumdis-2019-216563 (PMC7034344; doi:10.1136/annrheumdis-2019-216563)
Supplement: Supplementary data [file annrheumdis-2019-216563supp001.pdf]

Supplementary materials

Supplementary experimental procedures

Subjects

All procedures were approved by the Animal Care and Use Committee of China Medical University (No. KT2018060) and strictly adhere to the recommendations in the Guide for the Care and Use of Laboratory Animals of the National Institutes of Health. C57BL/6J male mice between 8 and 14 weeks of age used in this study were obtained from Vital River Laboratory (Beijing, China). Animals were housed two to four per cage under conventional laboratory conditions (12 h/12 h light/dark cycle, 22 °C) with ad libitum access to food and water throughout the experiments. All behavioral testing and procedures were conducted during the light phase of the cycle. The experimenter handled the mice on alternate days during the week preceding the first behavioral test. Experimenters were blind to the mouse treatments during testing and behavioral scoring. All surgeries were performed under anesthesia, and all efforts were made to minimize animal suffering.

Stereotaxic surgery

In urethane (1.6 g/kg, i.p.) anesthetized mice, two stainless-steel guide cannulas (24-gauge, 6-mm) using stereotaxic apparatus (#DW-2000, Taimeng, Chengdu, China) were implanted into the right and left sides of the ventral hippocampus (vCA1) at the

following coordinates: 3.3 mm posterior to the bregma, 3.8 mm left and right sides of the midline, and 3.9 mm below the top of the skull. Using three screws and dental acrylic, the cannulas were fixed to the skull. A 29-guage, 6-mm stylet was inserted into each cannula to keep them patent prior to microinjection. At least 10 days were allowed for recovery from the surgery.

Intra-hippocampal injection

Intra-hippocampal microinjections of anti-P IgG, control IgG or vehicle (artificial cerebrospinal fluid) were performed using a 30-gauge injection needle attached to polyethylene tubing to a 1-μl Harvard Apparatus syringe pump system (Pump 11 Elite). The volume of the solution to be microinjected into each ventral hippocampus was 0.5 μl and the microinjection was performed at the rate of 0.4 μl/min. For facilitating diffusion of the drug, the injection needle was left in place for a further 5 min after completion of microinjection.

Olfactory ability test

Mice were individually tested for time spent sniffing sequential presentations of different odors: water, two nonsocial odors (ie almond extract and vanilla extract), and two social odors as similarly reported previously (1). The test was performed on a separate cohort of mice at the same time-point at which the social recognition test would

have been performed to verify their olfactory abilities after administration.

Locomotor activity test

Mice were tested in an experimental apparatus consisting of an open plexiglass arena (40 × 40 × 40 cm) with even, overhead red-light illumination. Each session was video-recorded using an overhead digital camera. Each mouse was monitored for its locomotor activity in the empty open-field boxes for 1 hour.

Supplementary results

Intra-hippocampus injection of anti-P IgG did not affect general olfactory and locomotor abilities

Because olfaction is crucial for normal social interaction (2), we examined whether anti-P IgG injection influenced the detection or recognition of non-social or social odors (Fig.S1). The mice spent more time sniffing the social odors as compared with the nonsocial odors (two-way ANOVA with Bonferroni posttest, $p<0.0001$). But there was no significant difference of sniffing time across the anti-P, control- and vehicle-treated groups (two-way ANOVA with Bonferroni posttest, $p=0.6461$). We therefore conclude that the deficit in social discrimination in the anti-P-treated mice was not due to a defect in sensing social or non-social odors.

Gross locomotor functions were assessed in an empty open-field arena. Fig. S2A show the measure of total distance traveled over the 1-hour test. All three groups of mice

decreased the total distance traveled over time, indicating normal habituation to the novelty of the open-field arena. There was no a significant difference across the groups received different treatments. For the measure of time spent being mobile (Fig. S2B), all three groups of mice showed a decrease in the time they were mobile over the 1-hour test. Also, no significant difference was found between different animal groups. Hence, these results indicate that the social behavioral deficits found in mice treated with anti-P-IgG were not dependent on alterations of olfactory abilities or gross locomotor activity.

Figure legends

Fig. S1: Olfactory abilities of mice treated by vehicle, control- or anti-P-IgG. Quantifications of duration when the mouse was sniffing different odours. All data are displayed as mean ± SEM. n=6 in each group.

Fig. S2: Gross locomotor activity of mice treated by vehicle, control- or anti-P-IgG. A) Total distance traveled in 10-min intervals during 1-h exposure to open field arena. B) Time mobile in 10-min interval during 1-h exposure to open field arena. All data are displayed as mean ± SEM. n=6 in each group.

Acknowledgments

This work was supported by the grants from National Nature Science

Foundation of China under grant (31671080 to LQ), Department of Science & Technology of Liaoning Province (2017225024 and 2018225111 to PY) and Department of Science & Technology of Shenyang (19-109-4-15 to PY).

Reference:

1. Papaleo F, Yang F, Garcia S, et al. Dysbindin-1 modulates prefrontal cortical activity and schizophrenia-like behaviors via dopamine/D2 pathways. Molecular psychiatry2012;17:85-98.
2. Brennan PA, Zufall F. Pheromonal communication in vertebrates. Nature2006;444:308-15.
