# Supplementary figures and images for: Rapamycin prevents the impairments of social recognition induced by anti-P antibody in a murine model
Source: Ann Rheum Dis. 2019 Dec 9;79(3):428–9. doi: 10.1136/annrheumdis-2019-216563 (PMC7034344; doi:10.1136/annrheumdis-2019-216563)

**Fig.S1**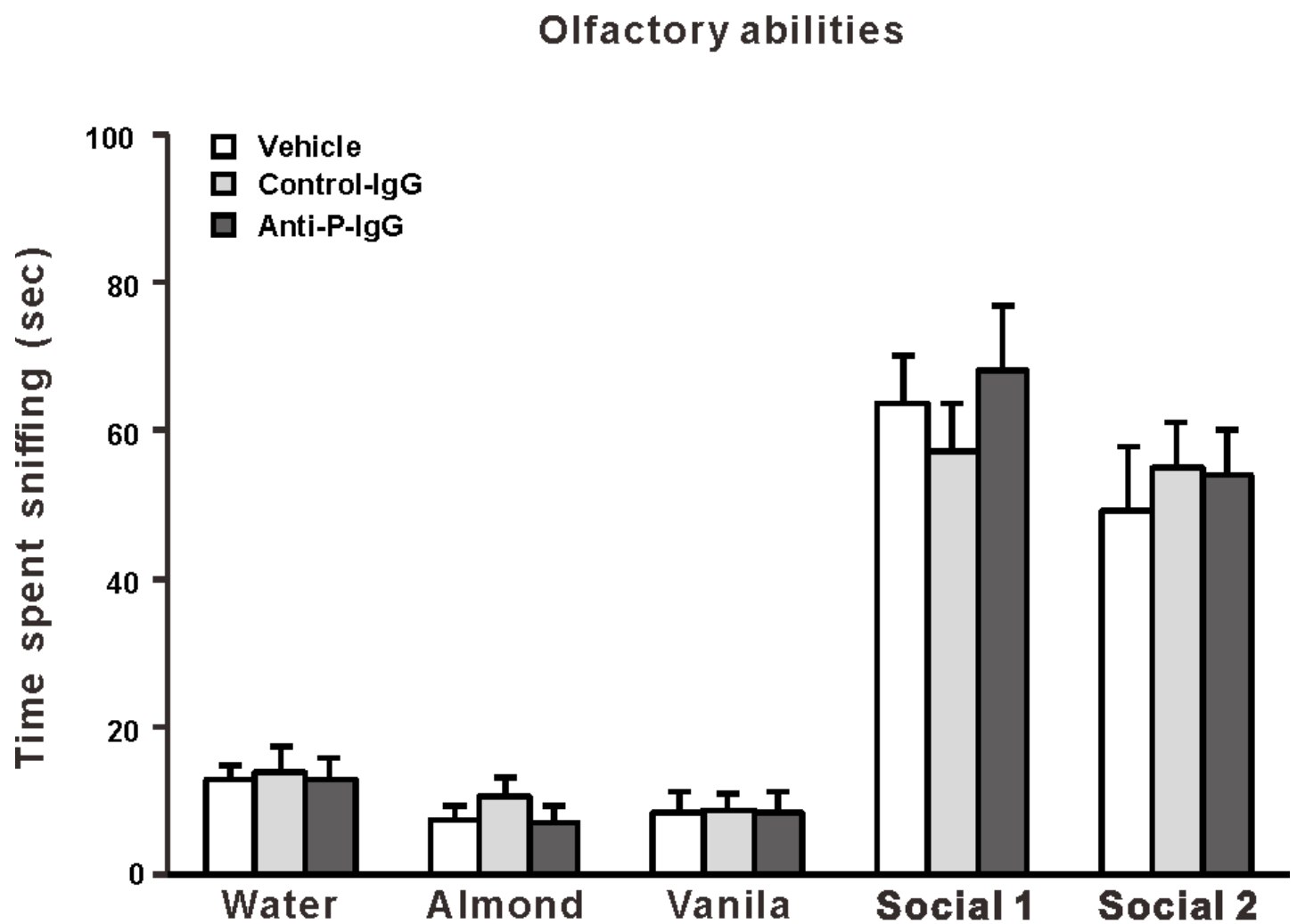

Supplement: Supplementary data [file annrheumdis-2019-216563supp002.pdf]

Fig.S2

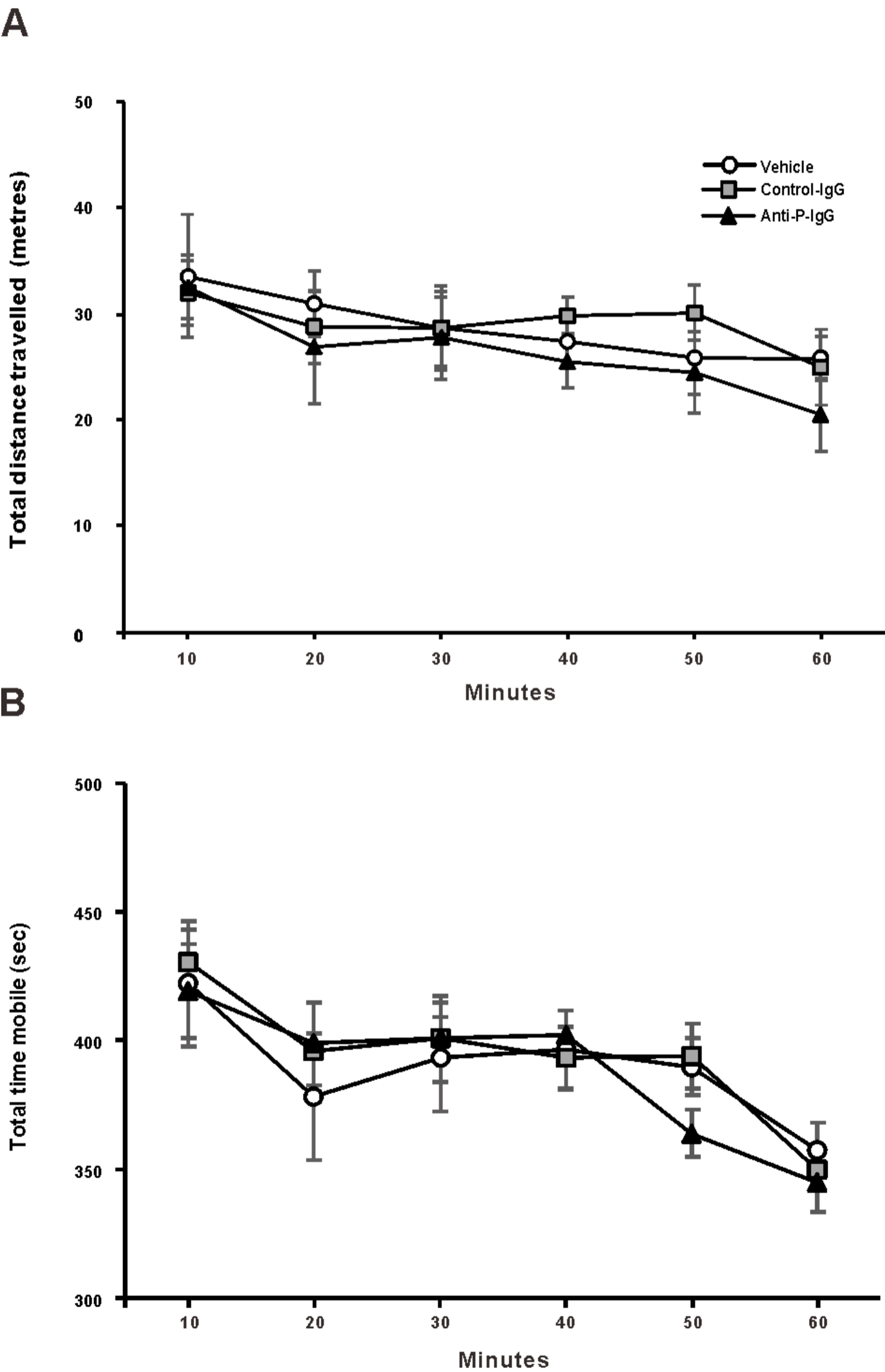

Supplement: Supplementary data [file annrheumdis-2019-216563supp003.pdf]
